# Recent Advances in Pediatric-Onset Multiple Sclerosis: From Early Diagnosis to Precision Care

**DOI:** 10.3390/children13070902

**Published:** 2026-07-07

**Authors:** Christian Lechner, Markus Breu

**Affiliations:** 1 Pediatric Neurology, Department of Pediatric and Adolescent Medicine, Medical University of Innsbruck, 6020 Innsbruck, Austria; 2 Department of Neurology, Medical University of Vienna, 1090 Vienna, Austria

Pediatric-onset multiple sclerosis (POMS) represents the earliest manifestation of chronic inflammatory demyelination of the central nervous system and accounts for only a small proportion of all multiple sclerosis (MS) cases [1,2]. Despite its rarity, POMS poses unique clinical challenges. Compared with adult-onset disease, children typically experience a highly inflammatory disease course with frequent relapses occurring during critical phases of neurological, cognitive, and psychosocial development [1,2]. At the same time, remarkable progress over the past decade has fundamentally transformed the field. Advances in neuroimaging, cerebrospinal fluid diagnostics, antibody testing, and disease-modifying therapies have improved diagnostic confidence and expanded therapeutic options, while increasing attention has been directed toward long-term outcomes, quality of life, and the transition into adulthood [1,2,3].

Reflecting these developments, the contributions assembled in this Special Issue provide a broad overview of current research in POMS, ranging from epidemiology and diagnostic biomarkers to treatment strategies, psychosocial aspects, and rare clinical presentations. Rather than addressing these topics in isolation, the studies collectively illustrate the ongoing evolution of pediatric neuroimmunology toward earlier diagnosis, individualized treatment, and comprehensive long-term care. For ease of reference, the articles included in this Special Issue are referred to as contributions throughout this editorial.

Understanding the epidemiology and disease burden of POMS remains fundamental to improving healthcare planning and clinical management [3,4]. Woo et al. (contribution 2) present nationwide epidemiological data from South Korea, highlighting the rarity of POMS while drawing attention to regional disparities in healthcare utilization and access to specialized care. Complementing these findings, Lechner et al. (contribution 10) provide the first regional epidemiological analysis of POMS in Tyrol, Austria, demonstrating one of the highest reported incidence rates in Europe and emphasizing the importance of structured referral pathways, comprehensive diagnostics, and timely treatment initiation. Extending the perspective beyond neurological disability, Tacke et al. (contribution 5) examine long-term educational achievement, socioeconomic participation, quality of life, and resilience in adults with childhood-onset MS. Together, these studies illustrate that understanding POMS requires not only accurate epidemiological surveillance but also an appreciation of its lifelong clinical and societal consequences.

Equally important is the continuing refinement of diagnostic precision. Early differentiation between monophasic acquired demyelinating syndromes (ADS) and chronic inflammatory disorders remains one of the greatest challenges in pediatric neuroimmunology [1,3]. Kauth et al. (contribution 4) provide novel evidence supporting the diagnostic value of the MRZ reaction in children with optic neuritis, suggesting that established biomarkers from adult-onset MS may also improve risk stratification in pediatric populations. Likewise, Turco et al. (contribution 6) investigate early predictors of outcome across the spectrum of pediatric ADS, demonstrating the prognostic value of integrating clinical, radiological, laboratory, and neurophysiological findings from the earliest stages of disease. Complementing these studies, Dică et al. (contribution 9) describe two children with Baló-type multiple sclerosis, illustrating the diagnostic complexity of tumefactive demyelinating lesions and emphasizing the importance of advanced neuroimaging, careful differential diagnosis, and multidisciplinary assessment. Collectively, these contributions reflect an important shift from establishing a diagnosis alone toward predicting disease evolution and enabling increasingly individualized clinical decision-making.

The growing role of biomarkers is particularly relevant in the broader field of pediatric demyelinating disorders. Longitudinal antibody testing, inflammatory blood parameters, and emerging serum biomarkers may contribute to the assessment of disease activity, relapse risk, and treatment response in selected contexts, although their clinical application still requires careful interpretation and further validation [5,6,7]. In this regard, the present Special Issue reflects a wider development in pediatric neuroimmunology: diagnostic and prognostic assessment increasingly relies on the integration of clinical phenotype, MRI, cerebrospinal fluid findings, serological markers, and longitudinal follow-up rather than on any single parameter alone [1,5,6,7].

Therapeutic advances constitute another major focus of this Special Issue. During the past decade, treatment of POMS has undergone profound changes, driven by the increasing availability of highly effective disease-modifying therapies and growing evidence supporting early intervention [8,9,10,11,12]. Walsh and Chitnis (contribution 1) provide a comprehensive overview of the rapidly evolving therapeutic landscape, discussing current disease-modifying therapies, emerging monoclonal antibodies, and contemporary treatment strategies, including escalation and induction approaches. Complementing this review, Eichinger et al. (contribution 7) evaluate therapeutic plasma exchange in pediatric neuroimmune disorders, demonstrating favorable clinical outcomes together with a well-characterized safety profile in children requiring escalation therapy. Together, these contributions illustrate how therapeutic decision-making has evolved from relatively limited treatment options toward increasingly personalized strategies that balance efficacy, safety, and long-term disease control.

Beyond controlling inflammatory disease activity, modern management of POMS increasingly recognizes the broader impact of the disease on affected children and their families. Aloni et al. (contribution 3) investigate illness perception in children with POMS and their parents, demonstrating that discrepancies in disease perception are associated with higher rates of anxiety and depression. These findings highlight the importance of addressing psychological well-being alongside neurological disease activity. Similarly, the long-term follow-up presented by Tacke et al. (contribution 5) demonstrates that although many individuals achieve favorable educational and neurological outcomes, quality of life may remain substantially influenced by pain, fatigue, anxiety, and depression. Consistent with these observations, health-related quality of life has become an increasingly relevant outcome in pediatric MS research and therapeutic trials [13]. Together, these studies reinforce the concept that successful treatment of POMS extends beyond relapse prevention and MRI stability to include psychological support, family-centered care, and optimization of long-term participation in education, employment, and everyday life.

The clinical heterogeneity of POMS is further emphasized by contributions addressing uncommon disease manifestations and challenging clinical scenarios. Dică et al. (contribution 8) explore the coexistence of epilepsy and POMS, suggesting that seizure disorders may identify a subgroup with more severe disease characteristics while also underscoring the potential contribution of independent genetic epilepsies. Complementing these observations, the accompanying case report by Dică et al. (contribution 9) further highlights the importance of multidisciplinary assessment and advanced diagnostic techniques when confronted with atypical inflammatory lesions. Although uncommon, these clinical presentations remind clinicians that POMS encompasses a remarkably diverse spectrum of disease requiring flexible and individualized diagnostic and therapeutic approaches.

Taken together, the contributions in this Special Issue illustrate the remarkable progress achieved in pediatric multiple sclerosis over recent years. Collectively, they reflect an evolving research landscape that extends beyond descriptive clinical characterization toward precision diagnostics, individualized therapeutic strategies, and comprehensive long-term care. Improvements in epidemiological surveillance, biomarker development, prognostic stratification, therapeutic innovation, and longitudinal follow-up are increasingly converging to support personalized management for children and adolescents with demyelinating diseases. Equally important, these studies underscore that further advances will depend on close collaboration among pediatric neurologists, adult neurologists, neuroradiologists, immunologists, psychologists, rehabilitation specialists, and basic scientists.

While important challenges remaining, including the identification of robust predictive biomarkers, optimization of treatment sequencing, prevention of long-term neurodegeneration, and facilitation of a seamless transition into adult care, the work presented in this Special Issue demonstrates the considerable progress already achieved. We hope that the studies presented here will stimulate further international collaboration, encourage multidisciplinary research, and ultimately contribute to improving the diagnosis, treatment, and long-term care of children and adolescents with pediatric-onset multiple sclerosis.

## Data Availability

Not applicable.
